# Outcome and characteristics of patients with adult grade 4 diffuse gliomas changing sites of treatment

**DOI:** 10.1007/s00432-022-04439-7

**Published:** 2022-11-08

**Authors:** Marie-Therese Forster, Marion Hug, Maximilian Geissler, Martin Voss, Katharina Weber, Maya Christina Hoelter, Volker Seifert, Marcus Czabanka, Joachim P. Steinbach

**Affiliations:** 1 Department of Neurosurgery, Goethe University Hospital, Schleusenweg 2-16, 60528 Frankfurt am Main, Germany; 2 University Cancer Center Frankfurt (UCT), University Hospital Frankfurt, Goethe University, Frankfurt am Main, Germany; 3 Department of Neurology, Goethe University Hospital, Schleusenweg 2-16, 60528 Frankfurt am Main, Germany; 4 Dr. Senckenberg Institute of Neurooncology, Goethe University Hospital, Schleusenweg 2-16, 60528 Frankfurt am Main, Germany; 5 Neurological Institute (Edinger Institute), Goethe University Hospital, Heinrich-Hoffmann-Str. 7, 60528 Frankfurt am Main, Germany; 6 German Cancer Research Center (DKFZ), German Cancer Consortium (DKTK), Heidelberg, Germany; 7 Department of Neuroradiology, Goethe University Hospital, Schleusenweg 2-16, 60528 Frankfurt am Main, Germany

**Keywords:** Second opinion, Referral, Adult diffuse glioma grade 4, Neuro-oncological center;, Secondary treatment

## Abstract

**Purpose:**

With increasing patient self-empowerment and participation in decision making, we hypothesized that patients with adult-type diffuse gliomas, CNS WHO grade 4 who change sites of treatment differ from patients being entirely treated in one neuro-oncological center.

**Methods:**

Prospectively collected data from all diffuse glioma grade 4 patients who underwent treatment in our neuro-oncological center between 2012 and 2018 were retrospectively examined for differences between patients having initially been diagnosed and/or treated elsewhere (External Group) and patients having entirely been treated in our neuro-oncological center (Internal Group). Additionally, a matched-pair analysis was performed to adjust for possible confounders.

**Results:**

A total of 616 patients was analyzed. Patients from the External Group (*n* = 78) were significantly younger, more frequently suffered from IDH-mutant astrocytoma grade 4, had a greater extent of tumor resection, more frequently underwent adjuvant therapy and experienced longer overall survival (all *p* < 0.001). However, after matching these patients to patients of the Internal Group considering IDH mutations, extent of resection, adjuvant therapy, age and gender, no difference in patients’ overall survival was observed anymore.

**Conclusion:**

The present study demonstrates that mobile diffuse glioma grade 4 patients stand out from a comprehensive diffuse glioma grade 4 patient cohort due to their favorable prognostic characteristics. However, changing treatment sites did not result in survival benefit over similar patients being entirely taken care of within one neuro-oncological institution. These results underline the importance of treatment and molecular markers in glioma disease for patients’ self-empowerment, including changing treatment sites according to patients’ needs and wishes.

## Introduction

With regard to optimizing quality and cost-efficiency in cancer care, dedicated neuro-oncological centers have been increasingly established worldwide during recent years. In Germany, 46 certified neuro-oncological centers had been listed by the German Cancer Society by December 31st, 2020, providing care for nearly 4000 brain tumor patients per year (Krebsgesellschaft 2021). Growing evidence exists on better outcome and longer survival of brain tumor patients in high-volume hospitals and academic centers (Aulakh et al. 2019; Hauser et al. 2018; Lopez Ramos et al. 2019; Raj et al. 2020; Trinh et al. 2015). Moreover, since curative treatment for glioma still is lacking, brain tumor patients are recommended to enroll in clinical trials, that are primarily conducted at academic centers and that have been shown to be linked to better survival (Hauser et al. 2018; Shahar et al. 2012; Tan et al. 2020). However, according to the pertaining literature and available data, only half of glioma patients are currently treated at academic or certified neuro-oncological centers (Hauser et al. 2018; Krebsgesellschaft, 2021; Lopez Ramos et al. 2019).

Data on patients being secondarily referred to one of these centers, either by regional institutions, where according specialists are not available, or upon patients’ requests, are scarce. To our knowledge, only one publication has investigated overall survival in patients with adult-type diffuse gliomas CNS WHO grade 4 with regard to care transition or treatment referral database so far, however, not taking patients’ characteristics into consideration (Zhu et al. 2019).

With the Internet being more and more used as a main source of information, as well as due to patients’ empowerment and their growing participation in shared decision making, the number of brain tumor patients seeking a second opinion also rises continuously (Elwyn et al. 2012; Tan and Goonawardene 2017). As could be shown for patients with malignancies of different origins, the need for reassurance of diagnosis and treatment recommendation, the wish to consider all options and opportunities and dissatisfaction with the first consultation are patients’ main reasons for seeking second opinions (Olver et al. 2020; Ruetters et al. 2016).

Since all these aspects may equally apply to patients suffering from diffuse glioma grade 4, we hypothesized that patients with diffuse glioma grade 4 changing treatment sites differ from patients with diffuse glioma grade 4 being entirely treated in one neuro-oncological center. We therefore aimed at investigating whether baseline characteristics as well as outcomes differ between patients’ primarily undergoing diagnosis and treatment in our neuro-oncological center and patients being referred to or consulting our neuro-oncological center after diagnosis and/ or treatment outside our institution.

## Materials and methods

### Study design

The study was designed as a retrospective single-center study on patients with diffuse glioma grade 4 having been treated in our neuro-oncological center between January 2012 and December 2018. Thus, all gliomas had been classified according to the WHO Classification of 2016 (Louis et al. 2016). With regard to the 2021 WHO Classification of Tumors of the Central Nervous System (Louis et al. 2021), both IDH wildtype glioblastoma and grade 4 IDH-mutant astrocytoma were hereafter named diffuse glioma grade 4 for this publication.

Study approval was granted by the local ethics committee (reference number 04/09), and only data of patients who had given written informed consent of the scientific use of their data were included.

### Study population

In a first step, two cohorts of patients with diffuse glioma grade 4 were constructed. One cohort comprised all patients who underwent both diagnosis and treatment of diffuse glioma grade 4 in our neuro-oncological center (Internal Group). Within the second cohort, all patients with diffuse glioma grade 4 who had undergone either only histopathological diagnosis or both diagnosis and initial treatment of diffuse glioma grade 4 before consulting our neuro-oncological center were included (External Group).

In a second step, to overcome possibly resulting differences in sample sizes and patient characteristics, an additional matched-pair analysis was performed. Therefore, a group of patients was extracted from the Internal Group and matched to patients of the External Group regarding (1) IDH mutation status, (2) extent of tumor resection, (3) adjuvant tumor therapy, (4) patients’ age and (5) gender.

### Data collection

All data had prospectively been collected and entered in our institutional data base, from where all data of interest was retrieved for the present study.

Patients’ baseline characteristics, including patients’ demographics, their Karnofsky Performance Scale (KPS) Scores (Peus et al. 2013), data on molecular tumor markers, on surgical and adjuvant tumor treatment, and patients’ participation in clinical studies as well as the course of tumor disease including overall survival were analyzed.

Of note, for patients having undergone surgical tumor treatment, extent of tumor resection had been assessed on MRI within 72 h after surgery and determined to be complete, subtotal (when there was less than 10% of the original volume as residual tumor), or partial, if less than 90% of the original tumor volume were removed. For patients having been operated on outside our institution, extent of tumor resection had been determined in the same way by a board-certified neuroradiologist of our center. During the course of disease, all patients had regular clinical and radiological follow-up examinations, usually every 3 months. Disease progression was diagnosed according to the RANO criteria (Response assessment in neuro-oncology criteria) (Wen et al. 2010). Noteworthy, all patients’ cases were discussed in a multidisciplinary tumor board giving treatment recommendations pre- and postoperatively, prior to new adjuvant treatment as well as in case of radiological or clinical changes.

Regarding molecular tumor characterization, mutations of IDH were determined by immunhistochemistry or DNA methylation analysis. Information on the O6-methylguanine-DNA methyltransferase (MGMT) promoter status was assessed by methylation specific PCR or by DNA methylation analysis.

### Statistics

Statistical analyses were performed with SPSS version 26 (IBM Corp., Armonk, NY). Data was analyzed using the Pearson chi-square tests and student’s t-tests as appropriate. Overall survival was estimated using the Kaplan–Meier method, and groups were compared using the log-rank test. A p-value < 0.05 was deemed statistically significant.

## Results

A total of 616 patients having been treated for diffuse glioma grade 4 in our institution between January 2012 and December 2018 were identified. Of these, 538 patients were initially diagnosed and treated in our neuro-oncology center, constituting the Internal Group, whereas 78 patients had undergone either histopathological diagnosis or both diagnosis and initial treatment before consulting our center, classifying them as belonging to the External Group.

### Comparison between groups

Comparing patient characteristics between the Internal and the External Group, several significant differences were observed (Table 1). First, patients of the External Group were significantly younger (55.9 ± 14.9 vs. 62.7 ± 13.5 years, *t*(89) = 4.380, *p* < 0.001). These patients also underwent significantly more frequently tumor resection (*X*^2^(3) = 28.525, *p* < 0.001) as well as adjuvant tumor treatment (*X*^2^(3) = 25.391, *p* < 0.001). Most strikingly, 51% of diffuse glioma grade 4 patients of the comprehensive Internal Group experienced only tumor biopsy compared to 18.2% of diffuse glioma grade 4 patients of the External Group, and the rate of diffuse glioma grade 4 patients without any adjuvant treatment came up to 20.6% in the Internal Group, whereas diffuse glioma grade 4 patients of the External Group all experienced further treatment. In terms of molecular markers, patients of the External Group harbored significantly more often IDH-mutant astrocytoma grade 4 and tumors with MGMT promoter methylation (7.8% vs. 3.3%, *X*^2^(2) = 99.028, *p* < 0.001 and 45.5% vs. 38.4%, *X*^2^(2) = 182.249, *p* < 0.001, resp.).

**Table 1:** Comparison of patient characteristics between groups

	Internal group	External group	*P*-value
Sample size, *n* (%)	538 (87.4)	78 (12.7)	
Gender, *n* female (%)	201 (37.4)	30 (38.5)	0.851
Age, years, median (range)	64.1 (5.4–88.5)	55.6 (23.2–87.7)	0.011
Preoperative KPS, median (range)	90 (30–100)	90 (60–100)	0.392
Extent of resection, *n* (%)			< 0.001
Gross total resection	96 (17.8)	34 (43.6)	
Subtotal resection	121 (22.5)	14 (18.0)	
Partial resection	49 (9.1)	15 (19.2)	
Tumor Biopsy	272 (50.6)	15 (19.2)	
Molecular markers			
IDH, *n* (%)			< 0.001
Wildtype	511 (95.0)	51 (65.4)	
Mutation	18 (3.3)	6 (7.7)	
No data	9 (1.7)	21 (26.9)	
MGMT-promoter methylation, *n* (%)			< 0.001
Methylated	207 (38.5)	35 (44.9)	
Unmethylated	1 (0.2)	25 (32.0)	
No data	330 (61.3)	18 (23.1)	
Adjuvant therapy, *n* (%)*			< 0.001
RT + TMZ	248 (46.1)	56 (71.8)	
Hypo-fx RT + TMZ	54 (10.1)	6 (7.7)	
Other	111 (20.6)	15 (19.2)	
None	111 (20.6)	0 (0)	
No data	14 (2.6)	1 (1.3)	
Study participation, *n* (%)	267 (49.6)	37 (47.4)	0.717
Overall survival, months; mean (SD)	14.3 (15)	21.1 (15.9)	< 0.001
Deceased, *n* (%)	407 (75.9)	60 (76.9)	0.848

*KPS* Karnsofky Performance Score; *IDH* Isocitrate dehydrogenase; *MGMT* O6-methylguanine-DNA methyl-transferase; *RT* radiotherapy; *TMZ* temozolomide; *hypo-fx* hypofractionated

**Table 2:** Baseline characteristics of matched groups

		Internal Group (*n *= 62), *n* (%)	External Group (*n* = 62), *n* (%)	Person Chi-Square	
				*X*^2^	*p*-value
IDH 1 (1st p.c.)	Mutated	6 (9.7)	6 (9.7)	0.000	1.000
	Wildtype	51 (82.3)	51 (82.3)		
	Unknown	5 (8.1)	5 (8.1)		
Extent of resection	Complete resection	27 (43.5)	28 (45.2)	0.149	0.985
(2nd p.c.)	Subtotal resection	12 (19.4)	11 (17.7)		
	Partial resection	10 (16.1)	11 (17.7)		
	Biopsy	13 (21.0)	12 (19.4)		
Therapy (3rd p.c.)	RT + TMZ	46 (75.4)	47 (77.0)	0.154	0.926
	Hypo-fx RT + TMZ	4 (6.6)	3 (4.9)		
	Other	11 (18.0)	11 (18.0)		
Gender (4th p.c.)	Male	39 (62.9)	36 (58.1)	0.304	0.714
	Female	23 (37.1)	26 (41.9)		

		Internal group, *n* ± SD	External group, *n* ± SD	Independent samples *t* test	
				*t*	*p* value
Age, years (5th p.c.)		55.8 ± 11.3	56.1 ± 14.2	– 0.108	0.914

*p.c.* parallelization criterion; *RT* radiotherapy; *TMZ* temozolomide; *hypo-fx* hypofractionated

**Table 3:** Differences between matched groups

		Internal Group, *n* (%)	External Group, *n* (%)	Person Chi-Square	
				*X*^2^	*p* value
MGMT	Methylated	32 (51.6)	31 (50.1)	52.609	0.271
	Not methylated	27 (43.5)	23 (37.1)		
	Unknown	3 (4.8)	8 (12.9)		
Study participation	Yes	36 (58.1)	32 (51.6)	0.521	0.470
	No	26 (41.9)	30 (48.4)		
Deceased	Yes	40 (65.6)	47 (75.8)	1.555	0.212
	No	21 (34.4)	15 (24.2)		

		Internal Group, mean ± SD	External Group, mean ± SD	Independent Samples *t* Test	
				*t*	*p* value
KPS, %		93.0 ± 11.4	93.1 ± 10.1	− 0.038	0.970
OS, months		22.0 ± 18.7	21.8 ± 15.4	0.086	0.931

**Table 4:** Reasons for changing treatment sites (*n* = 78)

	Number (%)
Patient’s wish, i.e. second opinion	54 (69.2)
Patient’s relocation	9 (11.5)
Emergency	5 (6.4)
Referral by external physician	3 (3.8)
No information available	7 (9)

**Table 5:** Individual patients’ timepoints within the course of disease before changing treatment sites (*n* = 78)

	Number (%)
Biopsy only	8 (10.3)
Resection followed by radiochemotherapy	37 (47.4)
Resection followed by radiotherapy	5 (6.4)
Resection followed by chemotherapy	1 (1.3)
Resection only	23 (29.5)
Tumor progression after finished therapy	4 (5.1)

**Fig. 1:**
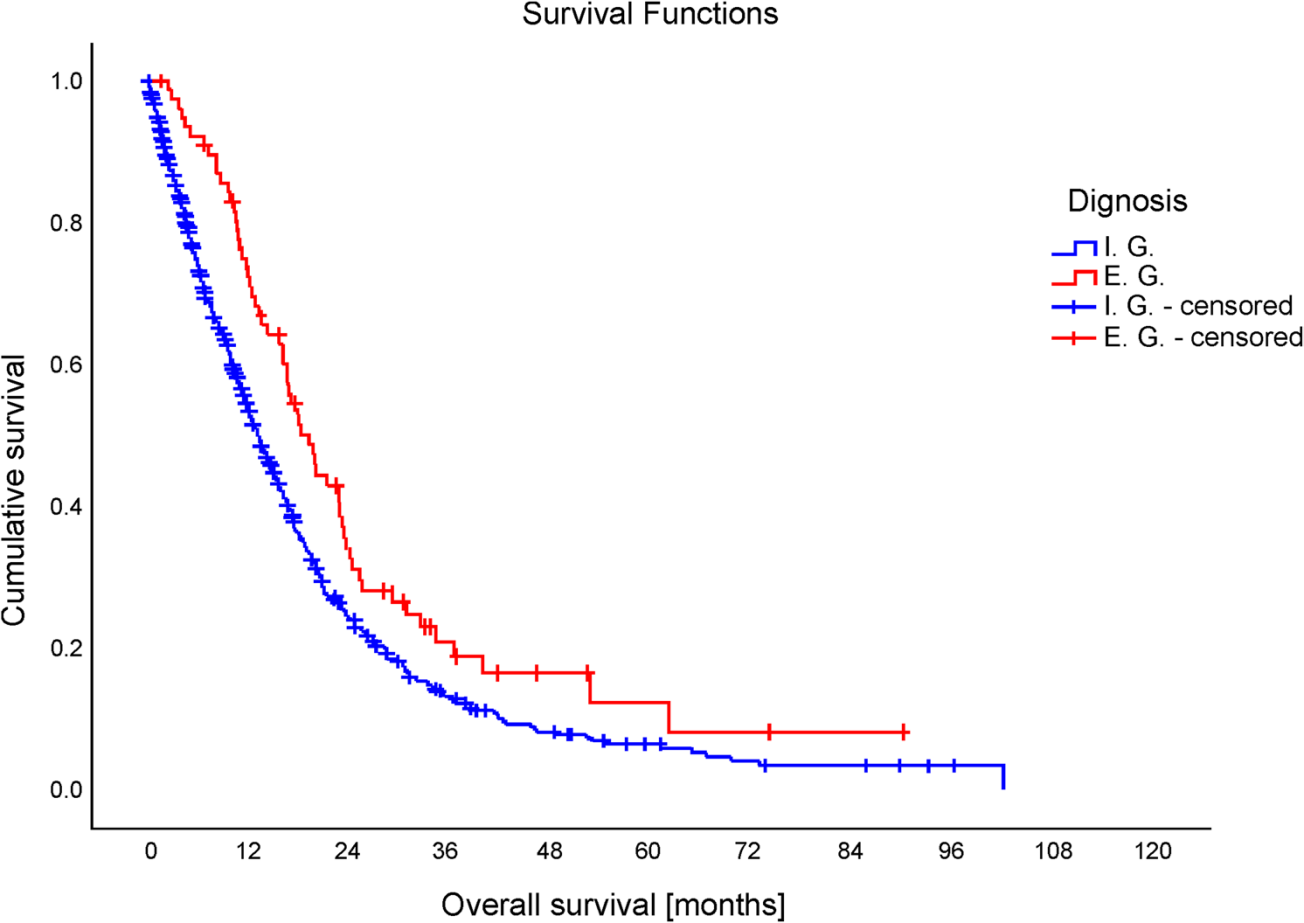
Mean overall survival of all patients. Patients of the External Group achieved a mean overall survival of 21.1 months compared with 14.3 months in patients of the Internal Group (t(98) = – 3.58, *p* < 0.001)

As of October 2021, 75.7% and 76.6% of patients of the Internal and External Group, respectively, had deceased (*X*^2^(1) = 0.037, *p* = 0.848), with patients of the External Group having achieved a mean overall survival of 21.1 months compared with 14.3 months in patients of the Internal Group (*t*(98) = – 3.58, *p* < 0.001; Log-rank test: *X*^2^(1) = 8.235, *p* = 0.004; for Kaplan–Meier curve see Fig. 1).

No statistically significant differences between Groups were found regarding the Karnofsky Performance Scale and regarding rates of study participation.

### Matched-pair analysis

To overcome not only differences in sample size but also significant differences of baseline characteristics between groups, patients of the Internal Group were matched by the IDH mutation status, extent of tumor resection, adjuvant therapy, gender and age in the respective order to patients of the External Group. Of note, equalizing for the IDH mutation status and extent of resection reduced respective sample sizes to 62 patients, due to missing information on the IDH mutation status in 21 patients of the External Group, but only 9 patients of the Internal group, with only 5 of these 9 patients presenting a comparable extent of tumor resection. Baseline characteristics of these two cohorts are listed in Table 2.

**Fig. 2:**
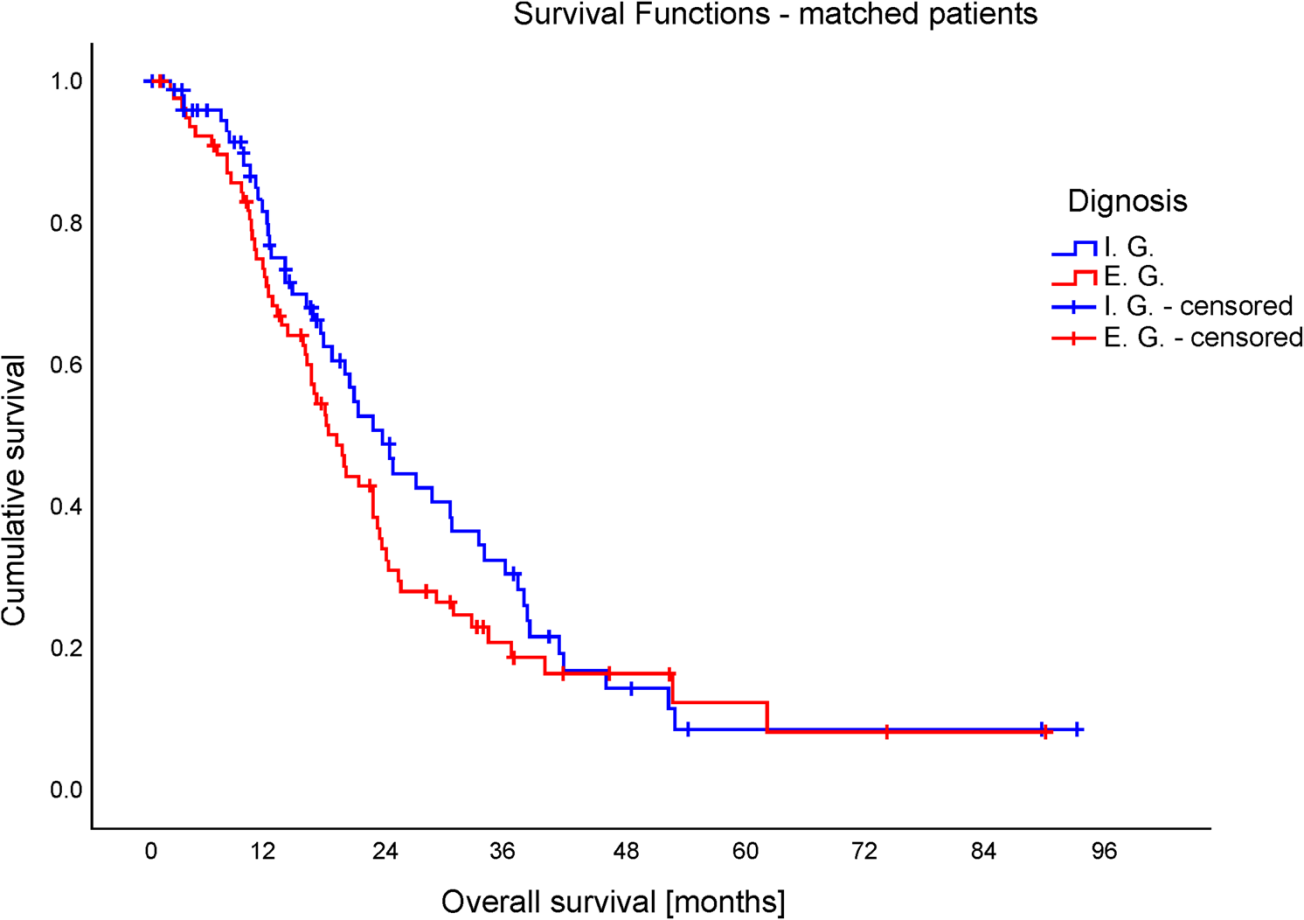
Mean overall survival of matched patients. Patients of the Internal Group achieved a mean overall survival of 22.0 months, comparable to mean overall survival of 21.8 months of patients of the External Group (*t*(122) = 0.086; *p* = 0.931)

As a result, no survival differences in matched groups were observed (*t*(98) = 0.086; *p* = 0.931; for Kaplan–Meier curve see Fig. 2), with slightly more patients of the External Group having deceased at the time point of observation (Log-rank test: *X*^2^ = 1.555, *p* = 0.212).

### Reasons for changing site of treatment

Exploring why patients changed sites of treatment, possible reasons were classified into patient’s wish (i.e. second opinion or dissatisfaction with previous treatment), patient’s relocation, referral by previous institution or an external physician (i.e. because of complexity of treatment or suggested study participation) and emergency referral (i.e. seizure or rapidly worsening clinical status).

An explanation for changing site of treatment was documented in all but 7 patient files, resulting in available data on 71 patients. Of these, 54 (69.2%) patients changed site of treatment on their own motivation, i.e. to seek for a second opinion, while patients’ relocation, referral on emergency or referral by an external physician were named reasons for changing site of treatment in 9 (11.5%), 5 (6.4%) and 3 (3.8%) patients, respectively (Table 3). Of note, 23 of the 78 patients changing treatment sites had been treated in another neuro-oncological center prior to treatment in our institution.

### Timepoint within the course of disease before changing treatment sites

All case files were examined to identify individual patients’ timepoints within their course of disease before changing treatment sites. Previous treatment was documented for all 78 patients (Table 4, 5).

In detail, 66 (84.6%) patients had undergone tumor resection prior to changing treatment sites, with 37 (47.4%) patients having thereafter additionally been treated by radiochemotherapy, 5 (6.4%) patients having thereafter undergone radiotherapy only and 1 (1.3%) patient having been treated by additional chemotherapy only. No adjuvant treatment had been administered to 23 (29.5%) patients after tumor resection and to 7 (9.0%) patients after tumor biopsy prior to their consultation of our center. Of note, only 4 (5.1%) patients changed treatment sites after external diagnosis of progressive disease.

## Discussion

This is the first study investigating possible differences in both patient characteristics and overall survival between patients with diffuse glioma grade 4 who changed their site of treatment and patients with diffuse glioma grade 4 who were entirely taken care of in one neuro-oncological center. A more favorable course of disease was observed for patients who changed site of treatment than for the comprehensive cohort of patients with diffuse glioma grade 4 having been entirely taken care of in one neuro-oncological center. However, after matching patients from both cohorts, no difference in overall survival was found. While our hypothesis—that patients who change sites of treatment stand out from the comprehensive cohort of patients undergoing their entire treatment in one neuro-oncological center—has thus been confirmed, it has, however, simultaneously, been refuted. Most importantly, the present results underline the significance of both treatment and molecular markers in glioma disease for patients’ self-empowerment, including changing sites of treatment during their course of disease.

Decades of research have led to a better understanding of the origin and clinical course of intrinsic brain tumors. Broad evidence exists, that extent of tumor resection significantly influences not only the course of high-grade glioma disease, but also patients’ overall survival (Marko et al. 2014; Molinaro et al. 2020). Moreover, in recent times, a number of molecular markers such as MGMT promoter methylation and IDH mutation status were identified as independent markers for diffuse glioma grade 4 patients’ outcome (Cohen et al. 2013; Eckel-Passow et al. 2015; Hegi et al. 2005; Weller et al. 2010). Accordingly, one of the major changes in the new 2021 WHO classification of Tumors of the Central Nervous System reflects the impact of IDH genes on the clinical–biological behavior of gliomas, with the type high-grade glioma now restricted to IDH-wildtype gliomas, whereas IDH-mutant gliomas with morphological hallmarks of high-grade glioma are now termed IDH-mutant astrocytoma CNS WHO grade 4 (Louis et al. 2021).

Likewise, better functional connectivity being associated with better neurocognitive performance has been observed in patients suffering from gliomas with IDH mutation compared to patients with IDH wildtype gliomas, although functional neuronal networks have been shown to be more often invaded by gliomas with IDH mutation than IDH wildtype glioma (Derks et al. 2019). This may suggest a relation of cellular rate of growth to molecular mechanisms of glioma cell integration along axonal projections or specific interactions with neuronal networks, both posing major limitations to maximize extent of tumor resection (Young et al. 2020).

However, in the present study, patients with diffuse glioma grade 4 who changed their site of treatment not only harbored more often tumors with IDH mutation but, most importantly, experienced more frequently higher extent of tumor resection. Thus, compared to 18% of corresponding patients in the External Group, half of the comprehensive Internal patient cohort underwent only tumor biopsy without further tumor resection. This observation entails two explanations, interpreting both, the relatively low number of tumor biopsies in the External Group of patients as well as the relatively high number of tumor biopsies in the Internal Group. First, patients undergoing only tumor biopsy mostly suffer either from tumors not amenable to tumor resection without harming or worsening patients’ neurological function or from tumors involving multiple brain regions. Many of these patients may already suffer from neurologic or neurocognitive impairments at the moment of tumor diagnosis, preventing them from seeking second opinion or changing site of treatment. Second, the relatively high proportion of tumor biopsies in relation to tumor resection within the Internal Group of patients reflects real world data in a large neuro-oncological institution. While patients with small or superficially located brain tumors might undergo tumor removal in decentral neurosurgical departments—diminishing the number of tumor resections in large comprehensive neuro-oncological centers—stereotactic or robot-assisted biopsies of brain tumors are often limited to large centers, where, moreover, diagnostic procedures and therapy are also offered to very old patients and patients with multiple co-morbidities, who might have rather experienced observation or best supportive care outside these institutions (Lorimer et al. 2016; Werlenius et al. 2020).

These considerations also apply to adjuvant therapy, which was found to differ significantly between the mobile cohort of patients and patients having been treated entirely in our neuro-oncological institution. Thus, both, the absence of any adjuvant therapy in 20.6% of patients as well as the relative frequent administration of hypo-fractioned radiotherapy combined with temozolomide (Perry et al. 2017) in the Internal Group might reflect the high number of older and surgically not amenable patients taken care of in large neuro-oncological institutions.

In contrast, patients who changed sites of treatment were younger, experienced more frequently tumor resection and all underwent adjuvant therapy, indicating more favorable patient and tumor characteristics. As could be shown by Hillen et al. (2017), younger patients are more likely to seek a second opinion and, as observed in the present study, more often move places for professional reasons. Interestingly, in the present study, most patients of the External Group changed sites of treatment after initial tumor resection followed by radiochemotherapy, indicating patients’ wish and need to be taken care of in a neuro-oncological center providing the highest level of quality cancer care.

Nevertheless, studies on seeking a second opinion are scarce and, to our knowledge, lacking for brain tumor patients. Whether changing sites of treatment influences glioma patients’ course of disease needs further evaluation. A longitudinal study on patients’ clinical and neurocognitive performance, their quality of life, treatment and outcome as well as on patients’ satisfaction with the quality of care will help to determine a possible impact of changing sites of treatment.

### Limitations

This study has several limitations. First, the large cohort of patients with diffuse glioma grade 4 in the Internal Group, who were entirely taken care of in one large neuro-oncological institution, comprised patients of any age in any clinical state and suffering from diffuse glioma grade 4 of any size and extension. In contrast, patients of the External Group were physically and mentally able to change sites of treatment, indicating a clear survivorship bias. Second, the overall difference in sample sizes as well as the difference in available data regarding both MGMT-promoter methylation and IDH mutation status did not allow for meaningful statistics, thus, the subsequent matched-pair analysis of patients was deemed the best approach to limit this mathematical imbalance. Consequently, further investigations on larger patient cohorts are warranted.

## Conclusion

The present data support our hypothesis that patients with diffuse glioma grade 4 changing sites of treatment differ from the comprehensive cohort of patients with diffuse glioma grade 4 being entirely treated in one neuro-oncological center. Patients changing sites of treatment are younger, frequently undergo a high extent of tumor resection followed by adjuvant therapy, often suffer from diffuse glioma grade 4 with favorable prognostic molecular profile, and, finally, experience better overall survival. The importance of tumor treatment and molecular markers for diffuse glioma grade 4 patients’ course of disease became evident after matching patients between groups according these characteristics, omitting any survival benefit.

Although this study could show that changing sites of treatment mostly results from patients’ motivation, further investigations on a larger number of patients, possibly via a multicenter approach, are necessary to elucidate the possible impact of their decision.

## Data Availability

Data of this work are available from the corresponding author upon reasonable request.

## Abbreviations

DNA: Desoxyribonucleic acid
IDH: Isocitrate dehydrogenase
KPS: Karnofsky Performance Scale
MGMT: O6-methylguanine-DNA methyltransferase
MRI: Magnetic resonance imaging
PCR: Polymerase chain reaction
RANO: Response assessment in neuro-oncology criteria
WHO: World Health Organization
